# Comparison of Four Polymerase Chain Reaction Methods for the Rapid Detection of Human Fecal Pollution in Marine and Inland Waters

**DOI:** 10.1155/2010/595692

**Published:** 2010-08-05

**Authors:** Dave S. Bachoon, Cortney M. Miller, Christen P. Green, Ernesto Otero

**Affiliations:** ^1^Department of Biological and Environmental Sciences, Georgia College and State University, Campus Box 81, Milledgeville, GA 31061-0490, USA; ^2^Department of Marine Sciences, University of Puerto Rico, Mayaguez Campus P.O. Box 9013, Mayaguez, PR 00681, USA

## Abstract

We compared the effectiveness of three PCR protocols for the detection of *Bifidobacterium adolescentis* and one PCR protocol for detecting *Bacteroidales* as indicators of human fecal pollution in environmental samples. Quantitative PCR indicated that a higher concentration of *B. adolescentis* DNA was recovered from sewage samples on the 0.2 *μ*m filters compared to the 0.45 *μ*m filters, and there was no evidence of qPCR inhibitors in the DNA extracts. With the Matsuki method (1999), *B. adolescentis* was detected only in undiluted sewage samples. The King method (2007) performed well and detected *B. adolescentis* in all of the sewage dilutions (from undiluted to 10^−4^). In contrast, the Bonjoch approach (2004) was effective at detecting *B. adolescentis* at lower dilutions (10^−3^) of sewage samples and it gave false positive results with some (3/8) pig fecal samples. Human-specific *Bacteroidales* (HuBacs) were detected in the lower diluents of sewage samples but was positive in pig (6/8) and cattle fecal samples. PCR detection of *B. adolescentis* in marine samples from Puerto Rico and freshwater samples from Georgia indicated that the PCR method of King et al. (2007) and the modified Layton method for HuBac were in agreement in detecting human fecal pollution in most sites.

## 1. Introduction

Fecal contamination can degrade the water quality in estuaries, beaches, lakes, and rivers to such an extent that these environments may become impaired for recreational, agricultural, and industrial uses. A major concern for resource managers is to determine the source of fecal pollution in order to apply appropriate corrective measures. In recent years, several molecular PCR-dependent approaches have been developed and used for detecting diagnostic sequences of the 16S rRNA gene of human fecal indicator bacteria as a marker for human fecal pollution. Many researchers use the amplicons from* Bifidobacteria *and *Bacteroidales *as molecular markers for indicating the presence of human fecal pollution [1–4]. While some studies have used the molecular detection of *Bifidobacterium adolescentis *to indicate the presence of human fecal pollution in environmental samples [1, 2, 4, 5], other researchers have relied on the detection of human-associated *Bacteroides *(HuBac) as a marker of human fecal pollution [6–8]. Currently, there are conflicting reports on which fecal bacterial group provides the most reliable marker for the presence of human fecal pollution in the environment. In addition, the lack of uniform methods of DNA extraction from environmental samples has added to the inconsistencies among reports on detection of human fecal pollution in the environment. 

DNA extraction from samples is a critical initial step in PCR detection of bacteria in environmental samples. There are numerous commercially available DNA extraction kits that are used for recovering bacterial DNA directly from feces, soil, and water samples [7, 9, 10]. Although the use of DNA extraction kits in research laboratories has become routine, the amount and quality of DNA recovered depend on the skill of the researcher and on the choice of the DNA extraction protocol. With the use of such kits, it is usually faster to recover DNA from fecal, sediment, and soil samples than from water samples. This is because the bacteria in a water sample are often concentrated using centrifugation or by membrane filtration prior to beginning the DNA extraction [1, 11, 12]. Typically, membrane filters of pore sizes of 0.2 *μ*m or 0.45 *μ*m are used for concentrating bacteria from water samples prior to DNA extraction [1, 2, 10]. There is limited information available in the scientific literature on which pore size of filters is the most effective in maximizing PCR detection of target bacteria from water samples. 

Bifidobacteria are strictly anaerobic, gram-positive rods that make up a significant portion of the intestinal microflora of humans and animals [13–15]. Certain species, such as *B. adolescentis, *have been shown to be associated with human feces and have been used as an indicator of human fecal pollution [10, 12, 16]. Recent culture-dependent surveys also suggest the prevalence of *Bifidobacter* species other than *B. adolescentis* in feces of commonly reared animals [13]. However, there are reports indicating that *B. adolescentis* can be detected in nonhuman fecal samples [2, 17, 18]. There are three common PCR methods that are often used to detect *B. adolescentis* in environmental samples. Matsuki et al. [16] developed and successfully used 16S rRNA gene primers BiADO-1 and BiADO-2 to detect *B. adolescentis* directly from human fecal samples. Bonjoch et al. [12] demonstrated that *B. adolescentis* could be detected in human sewage samples using a nested PCR reaction that first amplifies a 1.35 kb 16S rRNA gene fragment of the *Bifidobacterium* genus with the primer pair lm26 and lm3 followed by a second PCR with the primer pair Bi ADO-1 and BiADO-2. Later, an optimized nested PCR similar to the Bonjoch [12] method was developed, and it detected *B. adolescentis* in areas near human sources of fecal pollution in estuarine and freshwater environments [10, 11, 19]. The King [10] method, uses primers lm26 and 785R in the first PCR to produce a 756-bp product which is used as the template in the second PCR with the Bi ADO-primers. Previous reports have indicated positive correlations between the detection of *B. adolescentis* in marine and freshwater environments and fecal bacteria numbers [1, 3, 5, 11, 19]. However, the study by Lamendella et al. [18], using the one-step PCR protocol of Matsuki et al. [16], was unable to detect *B. adolescentis* in some sewage and human fecal samples, suggesting further variability in the detection of fecal pollution from human sources using *B. adolescentis* as a marker. 

These inconsistencies among methods for the detection of human fecal pollution in environmental samples have caused concern within the scientific community and resource managers. Currently, there is no information available on the comparison of PCR detection of *B. adolescentis* using the methods of Matsuki et al. [18], Bonjoch et al. [12], and King et al. [10], and of HuBacs in environmental samples. One aim of this paper was to determine the influence of membrane filters on the yield of DNA recovered from water samples and on subsequent PCR detection of fecal bacteria. Another aim of this paper was to evaluate these three common PCR approaches for detecting *B. adolescentis* and a single-step PCR approach for detecting HuBac as indicators of human fecal pollution in environmental samples. 

## 2. Material and Methods

### 2.1. Sample Collection

 Sewage samples from the City of Milledgeville municipal sewage treatment plant were serially diluted in 0.9% sterile saline solution to 10^−4^, and triplicate 100 mL samples were filtered through a sterile mixed cellulose 0.22-*μ*m-pore (Type GS, Millipore, Billerica, MA) or a 0.45-*μ*m-pore (GN-6-Pall Corporation, Ann Arbor,MI) membrane filter. Animal fecal samples from pig, chicken, cow, rabbit, and horse were collected from local farms in central Georgia. Freshwater grab samples (100 mL) were collected from Lacey Mill Road, Little River and Big Indian Creek in the Oconee watershed of middle Georgia and returned to the laboratory within two hours for processing. Marine water samples were obtained from eight sites close to public beaches in Puerto Rico, stored on ice, returned to the laboratory, and filtered through 0.22-*μ*m-pore nitrocellulose membrane filter (Type GS, Millipore, Billerica, MA). The filters from Puerto Rico were frozen and shipped on dry ice by overnight courier to Milledgeville, GA.

### 2.2. DNA Recovery

 Filters were processed with the MoBio Ultraclean Soil DNA Kit (Carlsbad, CA) using a modification of the “Alternative Protocol” given by the manufacturer [5, 10]. This involved separating the bead solution from the beads and placing it in a 15-mL centrifuge tube containing the filter. Solutions S1 and IRS were placed in the tube and vortexed vigorously for 15 minutes. The solution was removed from the centrifuge tube and placed in the bead tube. From this point on, the manufacturer's protocol was followed. DNA was extracted from animal fecal samples following the procedure of the MoBio Ultraclean Fecal DNA Kit. Extracted DNA was quantified, using a Nanodrop ND-1000 Spectrophotometer (Wilmington, DE), and visually inspected under UV light for integrity on a 1.5% agarose gel stained with ethidium bromide.

### 2.3. Evaluation of qPCR Inhibition Using an Exogenous Internal Control

 The DNA extracted from water and sewage samples was tested for the presence of PCR inhibitors by using salmon testes DNA from *Oncerhynchus keta* (Sigma, St. Louis, MO) as an exogenous internal control and known amounts of * B. adolescentis* genomic DNA ATCC number 15703D. The salmon testes DNA was diluted in sterile distilled and deionized water to create a standard curve ranging from 0.0068 ng to 68.4 ng of DNA. Quantitative PCR contained 1 *μ*L water or sewage sample, and 6.84 ng salmon testes DNA as templates in a single 25 *μ*L qPCR, and primers specific to the rRNA transcriber region 2 of *O. keta* [10]. Samples with mean Ct values within 3 standard deviations of the 6.84 ng DNA containing control standard were considered uninhibited as in King et al. [5]. In addition, samples were also amended with two different amounts of *B. adolescentis* DNA (0.5 and 5 ng) per qPCR reaction using the qPCR conditions stated below (Section 2.4) to evaluate possible discrepancies among filters and if effects of impurities on different concentrations of target were present. The changes in Ct values between amended and unamended samples were compared to evaluate if shifts in Ct values were proportional to concentration of amended *B. adolescentis* DNA as an indication of the presence of impurities in the DNA extracts.

### 2.4. QPCR Estimation of *B. adolescentis*

 Quantitative PCRs were prepared in 200 *μ*L optical tubes with the following components and then adjusted to a final volume of 25 *μ*L : 12.5 *μ*L Stratagene (La Jolla, CA) FullVelocity SYBR Green QPCR Master Mix, a 2× concentrated mixture of archaeal DNA polymerase, dNTPs (GAUC), stabilizers, neutralizing hot start monoclonal antibodies, a thermostable accessory protein, 1.5 mM MgCl_2_; 0.1 *μ*M of each primer, and 10 ng template DNA. The reactions were monitored in a MJ/MiniOpticon Real Time PCR Detection System (BioRad, Hercules, CA), under the following conditions: 95°C for 8 minutes; 40 cycles of 95°C for 20 s, annealing temp (Table 1) for 45 s, followed by melting curve analysis at 45–95°C every 1°C for 10 s. Cycle threshold (Ct) was determined automatically on the MiniOpticon following manual adjustment of the threshold fluorescence. *B. adolescentis* DNA ranging from 0.005 ng to 5 ng was used for the standard curve. All standards were run in duplicate, and all samples and controls were run in triplicate. A no-template control, containing all the PCR reagents without DNA, was run with each reaction. 

### 2.5. PCR Detection of *B. adolescentis*

**Figure 1:**
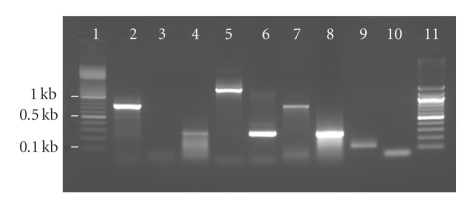
PCR results from water extracts used for various methods. Lane 1: 100 bp DNA ladder, Lane 2: B. Adolescentis positive control, Lane 3: negative control, Lane 4: Matsuki et al. [16], Lane 5: Bonjoch et al. [12]; first round, Lane 6: Bonjoch et al. [12]. Second round, Lane 7: King et al. [10]. First round, Lane 8: King et al. [10]; second round, Lane 9: Layton et al. [7], Lane 10: Layton et al. negative control, Lane 11: 100 bp DNA ladder.

 Prior to the PCR detection of Bifidobacteria, DNA samples were subjected to PCR using eubacterial primers 8F and 785R [20] to establish that the DNA recovered was suitable for PCR amplification. Three PCR protocols were followed to detect *B. adolescentis* in environmental samples using *B. adolescentis* genomic DNA ATCC number 15703D as a positive control. Primer sequence and annealing temperatures for each PCR method are listed in Table 1. The first approach was described by Matsuki et al. [16]. Another method used to detect *B. adolescentis* was the nested PCR approach as described by Bonjoch et al. [12] which was used to generate a genus-specific amplicon of 1.35 kb using primers lm26 and lm3 [21] in the first reaction followed by using primers BiADO-1 and BiADO-2 in a second PCR to produce the 279-bp marker of *B. adolescentis *(Table 1). The final PCR protocol used was the nested PCR procedure of King et al. [10]. The first step consisted of an amplification using the primers, IM26F and 785R, as the template for a second PCR mixture and amplified using *B. adolescentis *species-specific primers BiADO1-BiADO2 (Table 1) added to a 50-*μ*L reaction mixture [10]. PCR was performed under the following conditions: initial denaturing at 94°C for 5 minutes; 45 cycles of 94°C for 30 s, 48°C for 20 s, 55°C for 20 s, and 72°C for 1 minute; final elongation at 72°C for 5 minutes [10] and carried out with a Techne TC-312 Thermal Cycler (Cambridge, UK). Products from all PCRs were analyzed by electrophoresis in a 2% agarose gel stained with ethidium bromide and viewed in a gel documentation system to detect the presence of the appropriate bands as shown in Figure 1. 

### 2.6. PCR Detection of HuBac

 Human-associated HuBacs were detected using the primers HuBac566f and HuBac692r (Table 1). Each PCR reaction had a 50-*μ*L volume containing 0.3 mM dNTP, 3 mM MgCl_2_, 1 U Taq DNA polymerase, and 1× PCR reaction buffer. The samples were run on a Techne TC-312 Thermal Cycler under the following conditions: initial denaturing at 94°C for 5 minutes; 30 cycles of 94°C for 30 s, 60°C for 30 s, and 72°C for 30 s; final elongation at 72°C for 5 minutes. DNA extracted from sewage samples was used as a positive environmental control. All PCR amplification reactions included one without template DNA as a negative control. 

### 2.7. Molecular Detection of Fecal Contamination in Samples with Known Levels of Standard Fecal Pollution Indicators

 Water samples collected from marine and freshwater sites were assayed for the presence of *B. adolescentis* and *Bacteriodales.* Fecal enterococci were enumerated using the Enterolert system, whereas numbers of total *E. coli *were determined using the Colilert system (IDEXX Laboratories, Westbrook, ME). 100 mL of undiluted water samples and samples diluted (10^−1^–10^−2^) with sterile distilled water were placed in sterile, 100-mL polystyrene bottles and mixed with manufacturer-supplied growth medium until dissolved. The contents of each bottle were poured into a sterile Quanti-Tray panel containing 97 wells and is heat sealed. Quanti-Tray panels for fecal enterococci enumeration were incubated at 41 ± 0.5°C; those for total coliforms and *E. coli* were incubated at 35 ± 0.5°C. The presence of fecal enterococci and *E. coli* in wells was determined by detection of fluorescence with UV light at 365 nm. A manufacturer-supplied table was used to convert the number of positive wells to most probable number (MPN) values.

## 3. Results

### 3.1. Quantity and Quality of the DNA Extracts

 There were no significant differences between the total DNA recovered from 100 mL of sewage samples that have been diluted from 10^−1^ to 10^−3^ and filtered through 0.22- and 0.45-*μ*m-pore membrane filters (Table 2). The evaluation of PCR inhibition in the DNA extracts amended with salmon testes DNA suggests negligible effects of background impurities since samples filtered through different pore sizes had similar Ct prior to DNA addition (average Ct = 24; data not shown). Furthermore, the Ct was proportional to the spiked *B. adolescentis* DNA (*P* ≤ .95, SNK Multiple Comparison Test). In summary, both 0.22-*μ*m and 0.45-*μ*m-pore membrane filters spiked with 5.0 ng of *B. adolescentis* DNA had similar changes in Ct values (8.94 and 9.16, resp.) while the additiion of 0.5 ng of *B. adolescentis* DNA elicited an increase of Ct values of 5.90 and 5.51. QPCR estimates of the amount of *B. adolescentis* DNA recovered from sewage using 0.22-*μ*m-pore membrane filters were in average higher than those from 0.45-*μ*m-pore membrane filters, especially considering dilutions of up to 10^−2^ (Table 2). 

### 3.2. PCR Detection of Fecal Bacteria in Sewage

 The molecular methods for detecting human fecal bacteria in environmental samples were initially evaluated and compared using municipal sewage DNA and animal fecal DNA samples. Detection of *B. adolescentis* as a marker of human fecal bacteria in sewage samples with a single PCR [16] showed positive bands in only 2 out of 3 raw sewage samples and was ineffective with diluted sewage samples (Table 3). To increase the sensitivity of detecting *B. adolescentis* in fecal samples, Bonjoch et al. [12] developed a nested PCR assay for *B. adolescentis*. Overall, this nested PCR detection method was more successful at detecting *B. adolescentis* in diluted sewage samples and detected *B. adolescentis* in over half of the sewage samples, but B*. adolescentis* was detected in 3 out of 8 pig fecal samples. Using the nested PCR method developed by King et al. [10], *B. adolescentis *was detected in 100% of the sewage samples but not in the animal fecal samples tested, suggesting good specificity for detection of human-derived fecal pollution. It was observed with the nested PCR approaches that in the first PCR the putative marker of *Bifidobacteria* was not always visible in samples that were positive for *B. adolescentis* after the second round of PCR. HuBacs were detected in 5 out of 24 sewage samples analyzed, in most of the pig fecal DNA samples, and cow and horse samples. None of the DNA from sewage samples recovered from 0.45-*μ*m-pore membrane filters showed positive bands for HuBac, and the detection of HuBac decreased in sewage samples with increasing dilution (Table 3).

### 3.3. Molecular Detection of Human Fecal Pollution in Aquatic Environments

 IDEXX fecal bacteria enumeration indicated levels of enterococci and *E. coli* ranging from undetectable to hundreds of thousands CFU (Table 4). Molecular detection of *B. adolescentis* in DNA samples recovered from marine and freshwater environments indicated that *B. adolescentis* was detected in 5 of the marine sites and in 3 freshwater sites using the method of King et al. [10] (Table 4). All of these 8 sites had elevated levels of at least one of the fecal-indicator bacteria, based on established water-quality standards for full body contact (USEPA, 2004). However, the King [10] method did not detect the presence of *B. adolescentis *at the Patillas station in Puerto Rico characterized by elevated numbers of both fecal indicator bacterial groups. Using the method of Matsuki et al. [16], *B. adolescentis* was not detected in any of the environmental samples while the method of Bonjoch et al. [12] detected *B. adolescentis* only in one freshwater site at Lacey Mill Road (Table 4). The HuBac marker was detected in three samples from the marine environment that had elevated levels of fecal-indicator bacteria and in one of the freshwater samples from Lacey Mill Road site 2.

## 4. Discussion

It is well established that the concentrations and quality of DNA recovered from water samples influence any subsequent PCR analysis of that DNA for the detection of specific bacteria [10, 19]. Currently, in microbial source tracking of fecal pollution, numerous methods are used for the recovery of fecal bacterial DNA from water samples. Often the water samples are filtered through membrane filters of pore sizes ranging from 0.2 to 0.45 *μ*m for concentrating the bacteria prior to DNA extraction [1, 2, 10]. Differences between the concentration of total sewage DNA recovered from 0.22-*μ*m-pore nitrocellulose membrane filter (Type GS) and that from 0.45-*μ*m-pore membrane filter (GN-6) were mostly not significant until reaching dilutions >10^3^ (Table 2). It is not clear why the yield of DNA at higher dilutions was lower using smaller pore size filters, however variability is ruled out as it was minimal for this set of observations. The similarity among different sample dilutions points to saturation of the adsorbing matrix of the kit used. That is, the DNA retention capacity of cartridges used from the kits was surpassed by most of the dilutions used. Even though there were no major differences in the concentration of DNA recovered from most of the filters, it is possible that the presence of PCR inhibitors in the DNA extracts could have differentially inhibited PCR or qPCR assays. 

The lack of qPCR inhibition in DNA extracted from environmental samples using 0.22-*μ*m or 0.45-*μ*m filters and the MoBio kit has been reported in previous studies [5, 19]. However, collecting bacteria on a 0.22-*μ*m-pore mixed cellulose membrane filter increased the amount of *B. adolescentis* DNA detected in diluted sewage samples compared to the detection of these bacteria in sewage samples filtered through a 0.45-*μ*m-pore membrane filter. In our case, it seems that *B. adolescentis* could pass through 0.45-*μ*m pores as our analysis shows an order of magnitude higher in detection by using 0.22-*μ*m pore size filters than by 0.45 *μ*m ones (Table 2). These results agree with previous observations that suggested using 0.22-*μ*m-pore membrane filter (Type GS) instead of 0.45-*μ*m-pore membrane filter (GN-6) for DNA extraction from environmental samples [10]. 

PCR detection of host-specific fecal-bacteria has become very useful in tracking the source of fecal pollution in environmental samples. However, common fecal-indicator bacteria, *E. coli *and enterococci, were not used in this study because there are no known strains of these bacteria that are limited to a specific host [8, 19]. Comparison of three PCR methods for the detection of the fecal bacteria *B. adolescentis* indicated that direct detection of *B. adolescentis* by a single-step PCR [16] was only effective for detecting the bacteria in raw sewage samples concentrated on a 0.22-*μ*m filters. These results were not surprising because the original method of Matsuki et al. [16] was developed for use with cell cultures and human fecal samples. Other reports have indicated that the detection of *B. adolescentis* in environmental samples required multiplex PCR [10–12]. In contrast, the nested PCR detection methods for *B. adolescentis* [10, 12] readily detected *B. adolescentis* in raw and diluted sewage samples (Table 3). The method of King [10] was the most reliable of the three methods for detecting *B. adolescentis* in sewage samples. The higher sensivity of the King [10] compared to the Bonjoch [12] method for detecting *B. adolescentis *in parallel DNA extracts was attributed to the amplicon in the first PCR being smaller (777 bp) than the amplicon generated by the Bonjoch (1350 bp) PCR assay (Figure 1). The HuBac PCR assay performed well with undiluted sewage samples but was prone to cross-reaction with animal fecal samples [7, 17]. However, the lack of specificity and sensitivity of the HuBac assay must have been derived from our modification of the original method that was designed for a qPCR assay with a fluorescent probe for one, which is more economical and simpler to execute based on nonquantitative PCR [1, 7]. In contrast, the second round of amplification based on King [10] did not detect *B. adolescentis* in any of the nonhuman sources of fecal contamination. This points out the high specificity of the King [10] assay to *B. adolescentis* and indicates that there is a higher cross-reactivity of the other methods tested. However, previous studies have indicated that *B. adolescentis* was detected in some animal fecal samples, particularly in pigs [17]. It is important that the source-tracking approaches should complement sampling with knowledge of activities within the watershed that may impact conclusions. 

 Analysis of marine and freshwater samples from Puerto Rico and Georgia with known concentrations of the fecal indicator bacteria, *E. coil* and *Enterococcus sp.,* indicated that the PCR method of King [10] and the modified Layton method for HuBac were in agreement in detecting human fecal pollution in most sites with elevated levels of *E. coli* and *Enterococcus sp*. In West Luquillo beach, there were high levels of fecal bacteria but only the method of King [10] indicated the presence of human fecal pollution. In contrast, the Bonjoch [12] assay detected *B. adolescentis* in only one site at Lacey Mill Road. The location and close proximity of some of these samples such as Patillas, Costa Azul Creek, and Tubo-DRNA to human activity supports the finding of human fecal bacteria in those samples. 

## 5. Conclusions

 Molecular source tracking methods are very useful for identifying the source of fecal pollution in environmental samples, however, attention should be placed on choosing the most appropriate PCR procedure and molecular marker in order to avoid misleading results. The reliable detection of *B. adolescentis* in environmental samples by conventional PCR (nonquantitative PCR) as an indicator of human fecal pollution requires multiplexing. The general good agreement of *Bacteriodales* and *B. adolescentis* detection from field samples suggests that both methods are suitable for detection of fecal contamination in the environments examined. More work is needed to underpin the use of fecal indicators as a sole proof of human sources of fecal contamination. As evidenced previously, the incidence of *B. adolescentis* detection is higher for a limited number of common animal sources, including human, helping pinpoint sources of fecal contamination. The discrepancies observed in this paper compared to other papers on the detection of *B. adolescentis* as a putative marker of human fecal pollution were attributed to differences in the methods of DNA extraction from environmental samples and differences in the PCR protocol used (e.g., primer sets and nested PCR). Combining traditional methods for enumerating fecal indicator bacteria and multiple-host markers from different bacterial groups should increase the reliability of results. It is strongly recommended that 0.2-*μ*m-pore filters be used when qPCR approaches are conducted.

## Figures and Tables

**Table 1:** Oligonucleotide primers (16S rDNA) used to detect fecal bacteria for each PCR assay.

Primer name and sequence (5′–3′)	Annealing temp (°C)	Product size (bp)	Method
lm26 (GATTCTGGCTCAGGATGAACG)	55	1350	Bonjoch et al. [12]
lm3 (CGGGTGCTI*C*CCCACTTTCATG)			

lm 26 (GATTCTGGCTCAGGATGAACG)	48	777	King et al. [10]
785R (CTACCAGGGTATCTAATCC)			

BiADO1 (CTCCAGTTGGATGCATGTC)	55	279	Matsuki [16]
BiADO2 (CGAAGGCTTGCTCCCAGT)			Bonjoch et al. [12]
			King et al. [10]

HuBac566f (GGGTTTAAAGGGAGCGTAGG)	60	116	Layton et al. [7]
HuBac692r (CTACACCACGAATCCGCCT)			

*Primer pairs lm26-lm3 and lm26-785R are genus-specific primers for Bifidobacteria.

Primer pair BiADO1 and BiADO2 is species-specific for *B. adolescentis*

HuBAC566f and HUBAC692r are specific for human associated *Bacteroides*

**Table 2:** DNA recovered and qPCR estimation of *Bifidobacterium adolescentis* in sewage sample DNA extracts from 0.22 *μ*m and 0.45 *μ*m of filters.

Sample (*n* = 3)	DNA extracted (avg. ng/*μ*L)		qPCR of *B. adolescentis* (avg. ng) ×10^−2^	
	0.22 *μ*m	0.45 *μ*m	0.22 *μ*m	0.45 *μ*m
Sewage dilution 10^−1^	14.00 ± 1.66	16.17 ± 1.30	2.418 ± 0.23	0.142 ± 0.12
Sewage dilution 10^−2^	17.50 ± 4.50	12.13 ± 0.92	1.154 ± 0.13	0.112 ± 0.09
Sewage dilution 10^−3^	17.83 ± 3.10	14.57 ± 3.32	0.856 ± 0.25	0.288 ± 0.08
Sewage dilution 10^−4^	6.17 ± 0.90	13.37 ± 1.35	0.738 ± 0.34	0.319 ± 0.11

**Table 3:** Detection of human fecal bacteria (*B. adolescentis* and HuBAC) PCR Products in environmental samples by multiplex- [10, 12] and single-PCR assays.

Sample (*n*)	Matsuki et al. [16]	Bonjoch et al. [12]	King et al. [10]	Layton et al. [7]
Raw sewage (3)	2	2	3	3
Sewage dilution 10^−2^ on .22 *μ*m filter (3)	0	2	3	2*
Sewage dilution 10^−3^ on .22 *μ*m filter (3)	0	1	3	0
Sewage dilution 10^−4^ on .22 *μ*m filter (3)	0	0	3	0
Sewage dilution 10^−1^ on .45 *μ*m filter (3)	0	3	3	0
Sewage dilution 10^−2^ on .45 *μ*m filter (3)	0	3	3	0
Sewage dilution 10^−3^ on .45 *μ*m filter (3)	0	1	3	0
Sewage dilution 10^−4^ on .45 *μ*m filter (3)	0	1	3	0
Pig (8)	0	3	0	6
Chicken (4)	0	0	0	0
Cow (2)	0	0	0	2*
Rabbit (4)	0	0	0	0
Horse (4)	0	0	0	1*

Detection limit was reached in 1/10,000 diluents of sewage.

*Very faint bands

**Table 4:** PCR-detection of *B. adolescentis* and HuBac in marine and freshwater samples.

Location	IDEXX	IDEXX	*B. adolescentis*			HuBac
	Enterolert (avg. MPN/100 mL)	Colilert (avg. MPN/100 mL)	Matsuki et al. [16]	Bonjoch et al. [12]	King et al. [10]	Layton et al. [7]
Marine						
Parguera este pueblo, costa	25.95	390	−	−	+	−
Tubo Roto DRNA	241900	24190000	−	−	+	**+**
Barrero beach	35.15	66.7	−	−	−	−
Steps Rincon (Tres Palmas)	0	49	−	−	−	−
West of Luquillo Beach	22539.5	46390	−	−	+	−
Costa Azul Creek	291550	383686.67	−	−	+	**+**
Patillas, close to Bathrooms	2621.25	2807.75	−	−	+	**+**
Patillas, to the east close to house drainage	772.5	2742.5	−	−	−	−
Freshwater						
Lacey Mill Road site 1	59.8	42	−	+	+	−
Lacey Mill Road site 2	62.9	524.7	−	−	+	**+**
Little River	31.35	214.95	−	−	+	−
Big Indian Creek	10.4	24.05	−	−	−	−
